# Identification of ActivinβA and Gonadotropin Regulation of the Activin System in the Ovary of Chinese Sturgeon *Acipenser sinensis*

**DOI:** 10.3390/ani14162314

**Published:** 2024-08-09

**Authors:** Huamei Yue, Huan Ye, Rui Ruan, Hao Du, Chuangju Li

**Affiliations:** Key Laboratory of Freshwater Biodiversity Conservation, Ministry of Agriculture and Rural Affairs of China, Yangtze River Fisheries Research Institute, Chinese Academy of Fishery Sciences, Wuhan 430223, China; yhuam@yfi.ac.cn (H.Y.);

**Keywords:** activin, ovary, gonadotropin, Chinese sturgeon

## Abstract

**Simple Summary:**

Activin is a dimeric growth factor with diverse biological activities in vertebrates. This study aimed to investigate the regulatory role of the *activin* signaling pathway in the ovary of cultured *Acipenser sinensis*. One *activinβA* subunit with a full-length cDNA sequence of 1572 base pairs was identified. Multiple sequence alignment and phylogenetic analyses indicated the conserved evolution of ActivinβA from mammals to fish species. Transcripts of *activinβA* were distributed ubiquitously in ovary and non-ovarian tissues. The in vitro human recombinant Activin A incubation stimulated not only the *activin* system-related gene transcriptions of *activinβA*, *follistatin*, its receptors *activinRIIA* and *activinRIIB*, and *smad2*, *smad3*, and *smad4*, but also the ovary development-related genes *cyp19a1a*, *erα*, and *erβ*. Gonadotropin activated *activin* signaling by recruiting *activinβA*, *follistatin*, *activinRIIA*, and *smad2*. These results were helpful for not only the molecular exploration of activin signaling in fish species, but also the ovarian maturation regulation of *A. sinensis*.

**Abstract:**

Activin is a dimeric growth factor with diverse biological activities in vertebrates. This study aimed to investigate the regulatory role of the *activin* signaling pathway in the ovary of the endangered, cultured sturgeon species *Acipenser sinensis*. One *activinβA* subunit was identified, with a full-length complementary DNA (cDNA) sequence of 1572 base pairs. Multiple sequence alignment suggested that ActivinβA shared high sequence identities with its counterparts in four other sturgeon species. Phylogenetic analysis indicated the conserved evolution of ActivinβA among vertebrates from mammals to fish species. Transcripts of *activinβA* were distributed ubiquitously in the liver, kidney, intestine, ovary, midbrain, hypothalamus, and pituitary, with the highest transcription found in the pituitary. In Chinese sturgeon ovarian cells, in vitro human recombinant Activin A incubation stimulated the *activin* system-related gene transcriptions of *activinβA*, *follistatin*, its receptors -*activinRIIA* and *activinRIIB*, and drosophila mothers against decapentaplegic proteins (*smads*) *smad2*, *smad3,* and *smad4*. Ovary development-related mRNA levels of *cyp19a1a* and aromatase receptors of *erα* and *erβ* were enhanced by Activin A or human chorionic gonadotropin (hCG) incubation. Furthermore, 15 IU/mL hCG treatment increased the transcription levels of *activinβA*, *follistatin*, *activinRIIA*, and *smad2*. This suggested that the *activin* system was functional for the regulation of ovary development in Chinese sturgeon, possibly under the regulation of gonadotropin, by recruiting *activinβA*, *follistatin*, *activinRIIA*, and *smad2*. These results were helpful for the molecular exploration of activin signaling in fish species, as well as the ovarian maturation regulation of *A. sinensis*.

## 1. Introduction

Activin belongs to the transforming growth factor β (TGFβ) superfamily, and was originally identified from follicular fluid as gonadal peptides that stimulate follicle-stimulating hormone (FSH) secretion in pituitary cells [1,2]. It is a dimeric glycoprotein composed of two β subunits, namely βA and βB subunits, and the dimerization of these two subunits leads to the formation of Activin homodimer (Activin A with βA:βA subunits or Activin B with βB:βB subunits) or heterodimer (Activin AB with βA:βB subunits) [3]. Activin signaling is mediated through specific cell surface Activin type II receptors (either ACTRIIA or ACTRIIB), which then recruit and phosphorylate Activin type I receptors (ACTRIB, also known as Activin receptor-like kinase 4 (ALK4)) [3]. Subsequently, these receptors activate the downstream drosophila mothers against decapentaplegic protein (SMAD) signaling cascade by promoting the phosphorylation of SMAD2 and SMAD3 and then forming heterotrimeric complexes with common SMAD4 [4]. These complexes finally translocate to the nucleus and modulate gene expression as transcription factors [5].

In vertebrates, *activins* and their receptors exhibit a widespread tissue distribution and act as autocrine/paracrine factors for the regulation of diverse physiological activities, including tissue differentiation [6,7], wound repair [8,9], bone metabolism [10], immune responses [11], local regulation of pituitary hormones [12], spermatogenesis [13], and folliculogenesis [14]. In fish, both βA and βB subunits of *activin* have been identified from several species and their distribution in reproductive tissues has been demonstrated [15,16,17,18,19]. The *activin* βA and βB subunitswere both expressed in the thecal cells of follicles in the rainbow trout *Oncorhynchus mykiss* [16]. Moreover, paracrine roles of *activin* in ovarian functions were largely reported in zebrafish *Danio rerio* [20,21,22] and goldfish *Carassius auratus* [23]. In zebrafish, both *activin* and its type IIA receptor were expressed in the ovary, and both recombinant goldfish Activin B and recombinant human Activin A had potent stimulatory effects on the final oocyte maturation [24,25]. Furthermore, the effect of Activin on the final oocyte maturation could be blocked by co-treatment with the *activin*-binding protein Follistatin [24,25].

Using the zebrafish model, studies showed that pituitary gonadotropins such as hCG (a homolog of luteinizing hormone LH in teleosts) had a positive regulatory effect on *activin* subunits, the type IIA receptor, and the *activin*-binding protein *follistatin* in both time- and dose-dependent manners in follicle cells [17,22,26]. Interestingly, Follistatin also suppressed hCG-induced zebrafish oocyte maturation, suggesting *activin* as a downstream mediator of hCG, which functioned specifically via the zebrafish LH receptor (Lhr) [27]. Series experiments in zebrafish demonstrated that gonadotropin and *activin* promoted oocyte maturational competence, and their stimulatory effects could both be suppressed by *follistatin* [26].

Chinese sturgeon *Acipenser sinensis* is a large-sized anadromous fish distributed in the Yangtze River and East China Sea and is now critically endangered [28]. The natural spawning activities of Chinese sturgeon were interrupted for three consecutive years (2017–2019), which caused its natural population to be on the verge of extinction [29]. Controlled propagation was successful in achieving better species conservation [30]. However, it is rather difficult for breeding females to reach final sexual maturation due to a long period of sexual maturity (14–26 years) and a reproduction interval of 2–7 years. Therefore, a limited population of female broodstocks is available for artificial propagation, which hampers the speed of species recovery. This study aimed to investigate the actions of gonadotropin and Activin on the oocyte development of Chinese sturgeon. The *activin βA* subunit was identified, and the sequence characterization and tissue distribution were further analyzed in Chinese sturgeon. In addition, in vitro incubation of recombinant Activin A or hCG with ovarian cells was performed to examine the transcriptional changes in *activin* signaling-related and oocyte development-related genes. These results should be meaningful for not only the molecular exploration of the *activin* system in fish species, but also for the artificial ovarian maturation regulation and species conservation of *A. sinensis*.

## 2. Materials and Methods

### 2.1. Experimental Fish and Sample Collection

The five-year-old, artificially propagated Chinese sturgeons (*A. sinensis*) (average body weight of 4.37 ± 0.5 kg and average whole length of 89.07 ± 10 cm) used in this study were cultured in Taihu station, Yangtze River Fisheries Research Institute, Chinese Academy of Fishery Sciences. All fish handling procedures were performed with the approval of the Animal Care and Use Committee of the Yangtze River Fisheries Research Institute, Chinese Academy of Fishery Sciences (ID number YFI2021YHM01). Efforts were made to alleviate the suffering of fish as much as possible.

Three female cultured Chinese sturgeons were anaesthetized with 0.05% MS222 (Sigma, Shanghai, China) and decapitated. Since the sturgeons were to be sacrificed, the number of Chinese sturgeons used was limited to three for the purpose of species resource conservation. Partial tissue samples of the liver, spleen, kidney, intestine, ovary, midbrain, hypothalamus, and pituitary were quickly dissected and preserved in the RNAlater solution (Ambion, Austin, TX, USA). Samples were stored at 4 °C for 16 h, and then preserved in an ultralow freezer at −80 °C until the preparation of RNA for tissue distribution analysis. Another small piece of ovary was fixed in Bouin’s solution for histological analysis. The rest of the ovary tissue was used for the subsequent in vitro culture experiment.

### 2.2. Histological Analysis

The ovary tissue fixed in Bouin’s solution was embedded in paraffin, cut at 8 μm, and stained with hematoxylin and eosin (HE). Images of sections were observed under a light microscope (BX-51, Olympus, Tokyo, Japan) equipped with a digital camera (DP-73, Olympus).

### 2.3. Full-Length cDNA Sequence Cloning of ActivinβA

Total RNA of the Chinese sturgeon ovary was extracted by the RNeasy Plus Mini Kit (Qiagen, Dusseldorf, Germany) with the manufacturer’s instructions. First-strand SMART cDNA was then amplified with the SMARTer^®^ RACE 5′/3′ Kit (Takara, San Jose, CA, USA) as described. Fragmented cDNA sequence of *activinβA* was retrieved from the ovary transcriptome database of Chinese sturgeon [31] and verified by PCR with the primer pairs of *activin*-F1/*activin*-R1 (Table 1). Subsequently, 5′ and 3′ RACE (rapid amplification of cDNA ends) together with two rounds of nested PCRs were applied to obtain the rest of the 5′ and 3′ partial sequences. For amplification of the 5′ end cDNA sequence, the first round of PCR was conducted using the first-strand SMART cDNA as the template and the primer pair of *activin*-R1/UPM (Universal Primer Mix; Table 1). The obtained PCR product was then used as the template for the second round of PCR with the primer pair of *activin*-R2/UPMS (Universal Primer Mix Short) (Table 1). The 3′-end cDNA sequence of *activinβA* was cloned similarly by two rounds of PCRs, with the primer pairs of *activin*-F1/UPM and *activin*-F2/UPMS (Table 1), respectively.

### 2.4. Sequence Analysis

The nucleotide and amino acid sequence identities were searched against the BLAST program (NCBI, http://blast.ncbi.nlm.nih.gov/Blast.cgi, accessed on 6 August 2021). Conserved domains were predicted in the Conserved Domain Database (NCBI, https://www.ncbi.nlm.nih.gov/cdd, accessed on 6 August 2021). Multiple amino acid sequence alignments were accomplished with the CLUSTAL X program (version 1.83) and refined with the GeneDoc software (version 2.7.0). The Mega (version X) software was applied for the phylogenetic tree construction using the Maximum Likelihood method based on the Poisson correction model and bootstrap setting of 1000 replicates. The Activin sequence of *Drosophila melanogaster* was set as the outgroup root. All the amino acid sequences analyzed were downloaded from the NCBI website. The GenBank accession numbers were as follows: *Homo sapiens* NP_002183.1; *Mus musculus* NP_032406.1; *Gallus gallus* NP_001383472.1; *Xenopus tropicalis* XP_002933452.1; *Acipenser ruthenus* RXM98718.1; *Huso huso* KAK6492157.1; *Anguilla rostrate* XP_064186952.1; *Scleropages formosus* XP_029105445.1; *Latimeria chalumnae* XP_006012178.1; *Lepisosteus oculatus* XP_006634550.1; *Polypterus senegalus* XP_039609328.1; *Polyodon spathula* XP_041100451.1; *Acipenser oxyrinchus oxyrinchus* KAK1173939.1; *Carassius auratus* XP_026093429.1; *Labeo rohita* RXN19312.1; *Clarias magur* KAF5892151.1; *Protopterus annectens* XP_043920742.1; *Drosophila melanogaster* AAL51005.1.

### 2.5. Tissue Distribution Analysis

After total RNA extraction from the above eight tissue samples preserved in RNAlater solution, reverse-transcribed cDNAs were obtained by methods described in the PrimeScriptRT reagent Kit with gDNA Eraser (Takara, Kusatsu, Shiga, Japan). Relative real-time PCR was performed for temporal tissue distribution analysis. The PCR was performed in a volume of 20 μL with SYBR green real-time PCR master mix (Takara, Otsu, Shiga, Japan) on a QuantStudio 6 Flex real-time PCR system (Applied Biosystems, Foster City, CA, USA). The amplification protocols were as follows: 1 min at 95 °C, followed by 40 cycles of 15 s at 95 °C, 15 s at 54 °C, and 15 s at 72 °C. The housekeeping gene *ef1α* (*ef1α*-rF and *ef1α*-rR; Table 1) was chosen as the internal control, as suggested in [32]. The PCR amplification efficiency of each primer pair including *activin*-rF/*activin*-rR was evaluated by a standard curve. All primers used in this study met standards of efficiency between 90 and 110% and R^2^ ≥ 0.99. All samples were analyzed in triplicate and the relative transcription levels were calculated with the 2^−ΔCT^ method.

### 2.6. Human ActivinβA and hCG Treatment with In Vitro Ovarian Cell Culture

The freshly dissected ovary tissue of Chinese sturgeon was minced using stainless steel scissors and washed three times in DMEM medium (FBS included, Gibco, New York, NY, USA). It was randomly dispersed into a 6-well cell culture plate for the recombinant human ActivinβA (R&D systems, Minnneapolis, MN, USA) and human gonadotropin hCG (Macklin, Shanghai, China) treatment, respectively. Three wells were treated as one group with the protein or hCG solutions.

The recombinant human ActivinβA was first dissolved into RNA-free sterile water at 10 μg/mL, and three graded concentrations of 50 ng/mL, 100 ng/mL, and 200 ng/mL were used for ovarian cells incubation [33]. The hCG powder was dissolved with RNA-free sterile water into 1300 IU/mL according to the manufacturer’s instructions. For ovarian cell incubation, 15 IU/mL hCG was applied [17]. After 12 h pre-incubation at 28 °C with 5% CO_2_, the medium was discarded, and the cells were washed twice and incubated with the medium (control) or medium containing ActivinβA or hCG for 6 h. Ovarian cells treated in each well were collected for total RNA extraction. The treatment time was confirmed based on our preliminary experiment.

Relative real-time PCR was conducted for related gene transcription analysis, as described above. The sequences of *follistatin*, *activinRIIA*, *activinRIIB*, and *smad4* for primer design were searched against the transcriptome data of Chinese sturgeon [31]. Primer sequences of *smad2*, *smad3*, *cypa19a1a*, *erα*, and *erβ* were acquired from the previous study [34].

### 2.7. Statistical Analysis

All data were presented as mean ± SD. In tissue distribution analysis, the data were assessed by using one-way analysis of variance (ANOVA) followed by Duncan’s multiple range tests with the software SPSS 22.0 (SPSS Inc., Michigan Avenue, Chicago, IL, USA). In the ovarian cells in vitro incubation experiment, independent samples Student’s *t*-test was used, and Levene’s test was applied for equality of variances. A probability (*p*) of <0.05 was considered statistically significant.

## 3. Results

### 3.1. Molecular Characterization of ActivinβA in Chinese Sturgeon

Histological analysis of the ovary tissue used in this study suggested that the oocytes were mainly in the cortical-alveolar stage (stage II) (Figure S1). The full-length cDNA sequence of *activinβA* (GenBank No. PQ118000) cloned from the ovary of Chinese sturgeon was 1572 bp, including a 206 bp 5′ terminal untranslated region (UTR), a 190 bp 3′ terminal UTR, and an open reading frame (ORF) of 1176 bp encoding a protein of 391 amino acids (aa). The deduced amino acids were predicted to contain conserved domains of the transforming growth factor beta (TGF-β) propeptide (51-258 aa, underlined) and the TGF-β-like domain found in Inhibin beta A chain (284-391 aa, boxed) (Figure S2).

**Figure 1:**
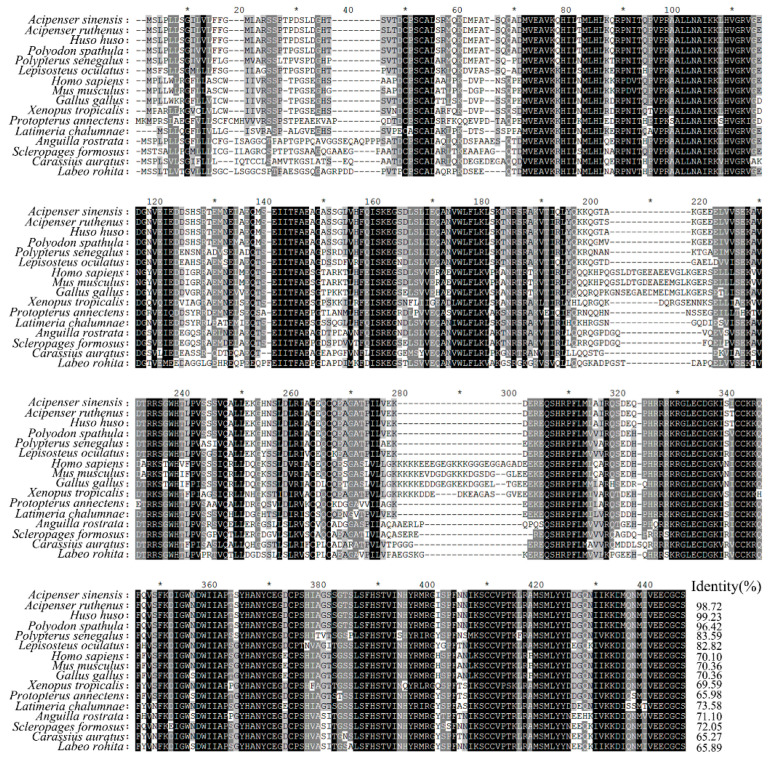
Multiple amino acid sequence alignment of ActivinβA of Chinese sturgeon with other representative vertebrates. Identical and similar amino acids are highlighted with black and gray shading. Identical amino acids were further marked with asterisks. Sequence identities are indicated at right.

**Figure 2:**
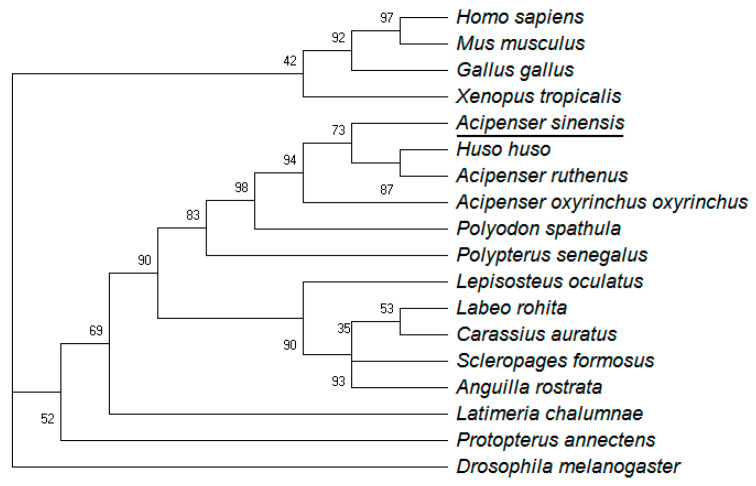
The Maximum Likelihood phylogenetic tree of Smad2/3 constructed by Mega X software of representative vertebrates. Horizontal branch lengths are proportional to estimated divergence of the sequence from the branch point.

Multiple amino acid sequence alignment showed that ActivinβA of Chinese sturgeon shared the highest sequence identity with that of *Huso huso* (99.23%), followed by that of the other two species in Acipenseridae, including *Acipenser ruthenus* (98.72%) and *Polyodon spathula* (96.42%) (Figure 1). Further phylogenetic analysis displayed that the analyzed vertebrate ActivnβA sequences formed two sub-clusters, including the tetrapod cluster and the teleost fish cluster (Figure 2). ActivinβA of Chinese sturgeon was situated in the teleost fish cluster and shared the same branch with another four sturgeon species: *Huso huso*, *Acipenser ruthenus*, *Acipenser oxyrinchus oxyrinchus*, and *Polyodon spathula*. The sturgeon branch was further clustered with the ancient fish species of *Polypterus senegalus*.

### 3.2. Tissue Distribution of ActivinβA in Chinese Sturgeon

**Figure 3:**
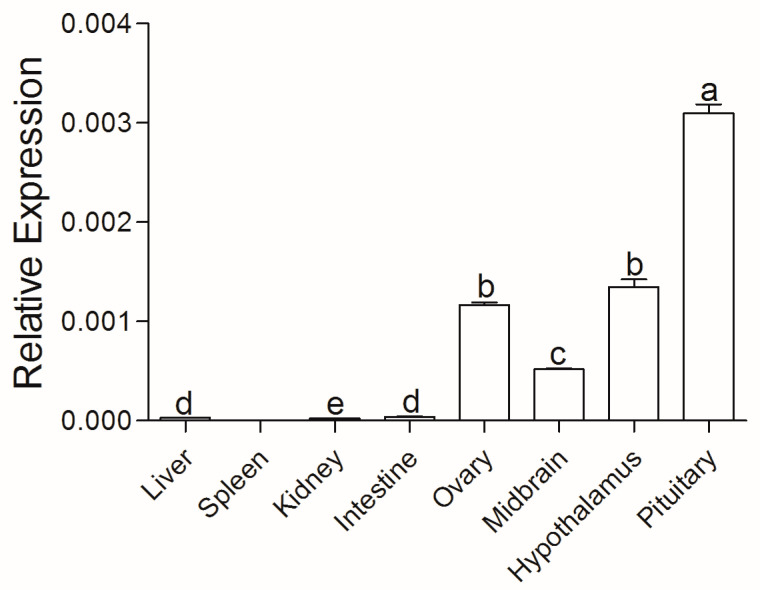
Tissue distribution analysis of *activinβA* evaluated by relative real-time PCR. Data are normalized to *ef1α* mRNA and represent mean ± SD of three separate experiments. Values with different letters above are significantly different (*p* < 0.05).

Relative real-time PCR analysis demonstrated that *activinβA* mRNAs of Chinese sturgeon were transcribed in liver, kidney, intestine, ovary, midbrain, hypothalamus, and pituitary tissues (Figure 3). The highest transcription levels of *activinβA* were present in the pituitary, followed by transcriptions in the hypothalamus and ovary.

### 3.3. Effect of Human ActivinβA on Activin Signaling Pathway-Related Gene Transcription

**Figure 4:**
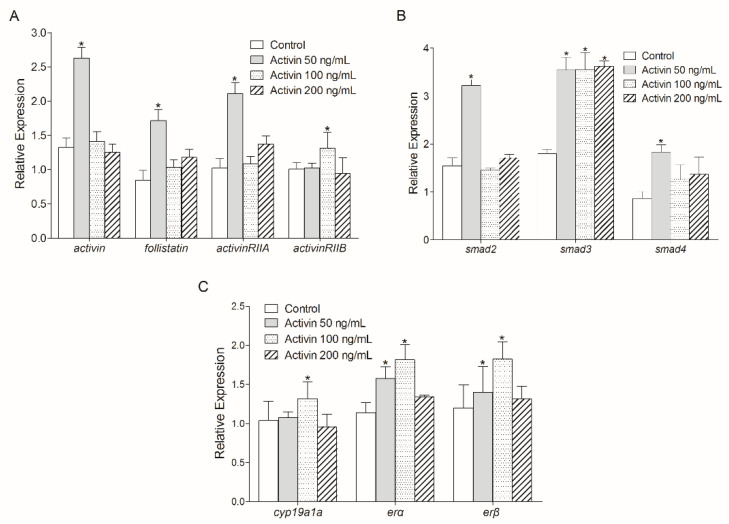
Effect of human Activin A incubation on the transcriptions of *activin*, *folistatin*, *activin* receptors (**A**), *smad* genes (**B**), and ovary development-related genes (**C**) in the ovarian cells evaluated by relative real-time PCR. Data are normalized to *ef1α* mRNA and represent mean ± SD of three separate experiments. Asterisks denote significant difference from control at *p* < 0.05.

Relative real-time PCR detection suggested that 50 ng/mL recombinant human ActivinβA protein incubation increased the mRNA levels of *activinβA*, *follistatin*, and *activinRIIA* in the in vitro ovary culture of Chinese sturgeon (*p* < 0.05) (Figure 4A). Additionally, *activinRIIB* transcription was significantly increased by 100 ng/mL ActivinβA treatment (*p* < 0.05) (Figure 4A). The transcriptions of three *smad* genes were also investigated, which showed that *smad3* transcriptions were all increased by the three doses of ActivinβA incubation (*p* < 0.05) (Figure 4B). However, increased mRNA levels of *smad2* and *smad4* were only exhibited in the 50 ng/mL ActivinβA treatment group (*p* < 0.05). Furthermore, 100 ng/mL ActivinβA led to the increase of *cyp19a1a* transcription, while mRNA levels of *erα* and *erβ* were both enhanced by 50 ng/mL and 100 ng/mL ActivinβA incubation, respectively (*p* < 0.05) (Figure 4C).

### 3.4. Regulation of Activin Signaling Pathway-Related Genes by Gonadotropin

**Figure 5:**
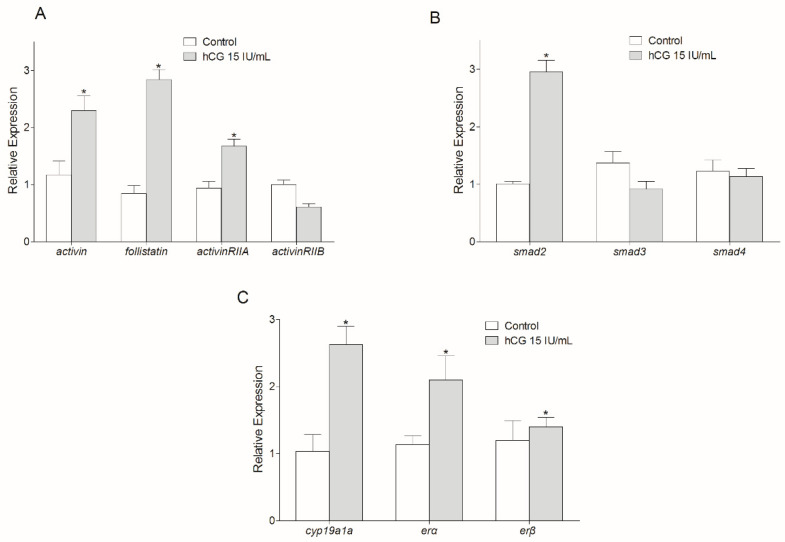
Effect of hCG incubation on the transcriptions of *activin*, *follistatin*, *activin* receptors (**A**), *smad* genes (**B**), and ovary development-related genes (**C**) in ovarian cells evaluated by relative real-time PCR. Data are normalized to *ef1α* mRNA and represent mean ± SD of three separate experiments. Asterisks denote significant difference from control at *p* < 0.05.

Treatment of the cultured Chinese sturgeon ovarian cells with hCG at 15 IU/mL caused a significant increase in the transcriptions of *activinβA*, *follistatin*, and *activinRIIA* (*p* < 0.05), with no significant change of the *activinRIIB* transcription (*p* > 0.05) (Figure 5A). The *smad2* mRNA level was increased by hCG incubation (*p* < 0.05), while no significant changes were found in the transcription levels of *smad3* and *smad4* (*p* > 0.05) (Figure 5B). Furthermore, hCG treatment led to significant enhancement of the mRNA levels of *cyp19a1a*, *erα*, and *erβ* (*p* < 0.05) (Figure 5C).

## 4. Discussion

In the present study, the β subunit of *activin A* in one primitive species of Chinese sturgeon was identified, and the sequence characterization suggested that it contained the typical conserved domains of the TGF β superfamily. The spatial tissue distribution and paracrine function of *activinβA* in the ovarian development regulation of Chinese sturgeon were investigated as well. The results of this study will help to provide a theoretic basis and technological support for both the ovary maturation regulation and species conservation of the endangered Chinese sturgeon.

The amino acid sequences for the Activin βA subunit were recorded in five sturgeon species, as well as in the primitive fish species *Polypterus senegalus* (Figure 2). This suggests that the Activin βA subunit was conserved throughout evolution from mammal to fish species, which also indicates its important and conserved role for physiological functions in vertebrates. Earlier studies of *activinβA* in fish species were limited to species such as zebrafish, goldfish, and rainbow trout; our research of *activinβA* in Chinese sturgeon enriched the molecular exploration of teleost *activin* subunits. Subsequent spatial distribution analysis showed that *activinβA* was transcribed extensively in the tissues, including pituitary and ovary, of Chinese sturgeon (Figure 3). This result is in accordance with previous studies, both in rodents [35,36,37] and fish models [15,38,39,40], revealing the wide expression of *activinβA* both in the ovary and in non-ovarian tissues, such as the pituitary, placenta, liver, different brain areas, etc. The diverse distribution pattern corroborates the common finding that *activin* mainly serves as an autocrine/paracrine factor rather than an endocrine hormone [41]. Furthermore, the highest transcription of *activinβA* in the pituitary of Chinese sturgeon might indicate the paracrine modulation of pituitary function for the *activin* system.

In goldfish, iodinated human Activin A is bound to ActRIIB-transfected cells, and this binding could be completely blocked by unlabeled Activin, indicating the specific affinity of human Activin with fish ActRIIB [42]. Human recombinant Activin A incubation also evaluated the *actRIB* mRNA levels in the pituitary cells of grass carp *Ctenopharyngodon idellus* [43]. Another report in tilapia has demonstrated that human Activin A stimulated the expression of glycoprotein hormone, FSH, and LH mRNAs in pituitary cells [44]. Therefore, it is evident that human Activin A was useful for probing the *activin* system mediated by Activin receptors in fish species. Herein, 50 ng/mL human Activin A incubation promoted ovarian transcriptions of *follistatin*, *activinRIIA*, *activinRIIB*, *smad2*, *smad3*, and *smad4* (Figure 4A,B), which indicated the existence of autocrine regulations of *activin* signaling systems in the ovary of Chinese sturgeons. Furthermore, Activin incubation stimulated the mRNA levels of *cyp19ala*, *era*, and *erβ*, which reinforces the ovary development regulatory role of the *activin* signaling pathway in Chinese sturgeon.

In previous studies of zebrafish, hCG upregulated Activin A protein expression [25] and *activin βA1* and *actRIIA* mRNA levels [17,22]. Furthermore, the Activin-binding protein Follistatin blocked hCG-induced oocyte maturation in zebrafish [24,25]. The stimulatory effect of ovarian *activin* by gonadotropin was consistent with the reports in mammals [45,46] and humans [47]. In Chinese sturgeon, hCG improved the transcription levels of *cyp19ala*, *era*, and *erβ*, suggesting its effective stimulation of ovary development. In addition, mRNA levels of *activinβA*, *follistatin*, *activinRIIA*, and *smad2* were upregulated by hCG incubation, while transcripts of *activinRIIB*, *smad3*, and *smad4* were not changed (Figure 5). This indicated that hCG stimulated ovary development by regulation of the *activin* system via recruiting *activinRIIA* and the downstream *smad2* in Chinese sturgeon.

In conclusion, the *activinβA* subunit was characterized in *Acipenser sinensis*, and spatial distribution analysis demonstrated its diverse transcription in tissues. The activin system was able to regulate ovary development in an autocrine way. Gonadotropin activated the activin system in the Chinese sturgeon ovary by increasing the transcription of *activin*, *follistatin*, its receptor *activinRIIA*, and the downstream factor *smad2*.

## Figures and Tables

**Table 1:** Primers used in this study.

Primer	Sequence (5′-3′)
*activin*-F1	ATGTCCTTGCCTCTGCTGAGTG
*activin*-F2	GTCCTCTGGCACCTCTCTCTCCTT
*activin*-R1	AGAGCAGCCACATTCCTCTACTATCA
*activin*-R2	CATTCTGGAGAGGGCACAAGAGGGG
UPM	CTAATACGACTCACTATAGGGCAAGCAGTGGTAT CAACGCAGAGT
UPMS	CTAATACGACTCACTATAGGGC
*activin*-rtF	GGACAGAGATGAATGAGTTGGCT
*activin*-rtR	AGCTTGAGAAAGAGCCAGACGT
*follistatin*-rtF	ACCAATAATGCCTACTGCGTGA
*follistatin*-rtR	TCCCTCGTATGCCACTCCTATC
*activinRIIA*-rtF	ATGTTGGTCTTGCCCTCTTCC
*activinRIIA*-rtR	AGGTCATCTTTCCCTGTTCGT
*activinRIIB*-F	GGATGCCTTCCTCAGAATAGA
*activinRIIB*-R	ATGTTTCAGCCAGCAGTCCTT
*smad4*-rF	AGGATTGACATTACAAAGTTCTGCT
*smad4*-rR	GACTCTGAAGGCGGTAGCG

## Data Availability

All data related to this study were presented in the manuscript.

## Supplementary Materials

The following supporting information can be downloaded at: https://www.mdpi.com/article/10.3390/ani14162314/s1, Figure S1. Histological analysis of ovary tissue by H.E. staining. Scale bar is 50 μm. Figure S2. Full-length cDNA sequence and deduced amino acids of *activinβA* in Chinese sturgeon. Nucleotides (upper line) are numbered from 5′ to 3′. The transforming growth factor beta (TGF-β) propeptide is underlined. The TGF-β-like domain found in Inhibin beta A chain is boxed. The asterisk (*) indicates the stop codon.
