# Supplementary material for: Identification of ActivinβA and Gonadotropin Regulation of the Activin System in the Ovary of Chinese Sturgeon Acipenser sinensis
Source: Animals (Basel). 2024 Aug 9;14(16):2314. doi: 10.3390/ani14162314 (PMC11350771; doi:10.3390/ani14162314)
Supplement: Supplementary file 1 [file animals-14-02314-s001.zip › animals-3080902-supplementary.pdf]

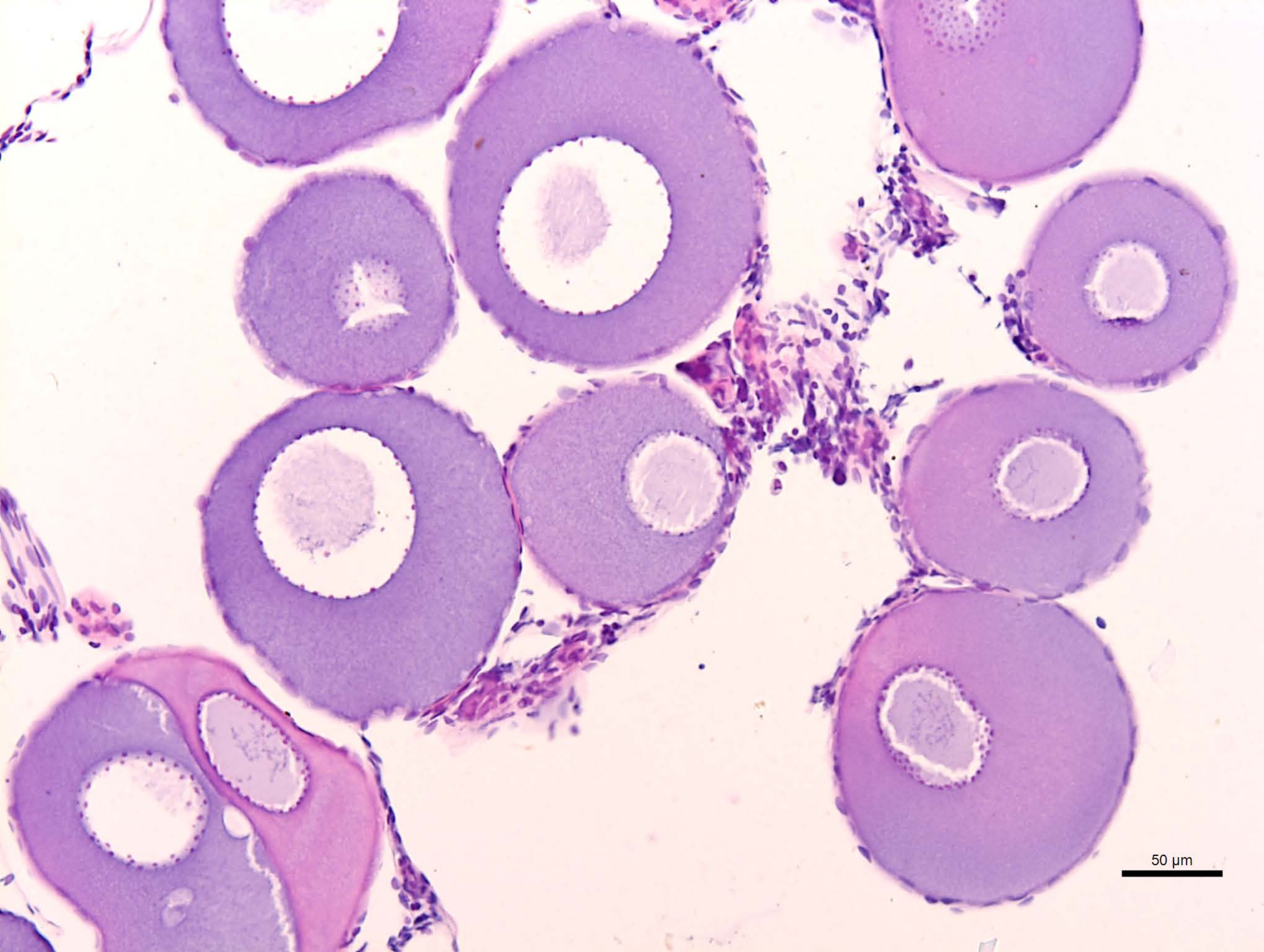
50 µm

```
1       TAGAGTAC
9       ATGGGGGCACATCCCTTCAGCACTTCTACACTCTCACGGACCTCTCCAGCTGACAAACTTTTTGCTAACAGACAGAAGACAGCAGAAAGTATTTTTTT
108     TAATTTAAAAAAGGGATTATTCTTTGTGGTTTGCTTAGTTTATTTTAATACCACCAAAAAGGGAAAACTTCATTTTAAGACCAACTCTACCCATCCAGG
207     ATGTCCTTGCCTCTGCTGAGTGGAATTCTGGTGCTCTTCTTTGGAATGCTGGCAAGGAGCTCACCGACCCCTGACTCCCTGGATGGGCACACATCTGTC
1        M  S  L  P  L  L  S  G  I  L  V  L  F  F  G  M  L  A  R  S  S  P  T  P  D  S  L  D  G  H  T  S  V
306     ACCGACTGCCCCTCTTGTGCCCTCTCCAGAATGCAAAAGGATATGCCGGCCACTTCCCAGCAGGCAGACATGGTGGAGGCAGTCAAGCAGCACATCTTG
34       T  D  C  P  S  C  A  L  S  R  M  Q  K  D  M  P  A  T  S  Q  Q  A  D  M  V  E  A  V  K  Q  H  I  L
405     ACCATGCTGCACTTGAAACAGAGACCCAACATCACCCAACCCGTGCCCAGGGCAGCCCTCCTGAATGCCATCAAAAAGCTGCATGTGGGCAGAGTTGGG
67       T  M  L  H  L  K  Q  R  P  N  I  T  Q  P  V  P  R  A  A  L  L  N  A  I  K  K  L  H  V  G  R  V  G
504     GAGGATGGCAATGTTGAGATTGAGGATGACAGCCACAGCCGGACAGAGATGAATGAGTTGGCTGAGCAAATGTCTGAAATCATCACTTTTGCTGAGGCA
100      E  D  G  N  V  E  I  E  D  D  S  H  S  R  T  E  M  N  E  L  A  E  Q  M  S  E  I  I  T  F  A  E  A
603     GGTGCCTCGTCAGGCTTGGTGCACTTTCAGATCTCCAAGGAGGGCAGCGACCTTTCACTCATAGAGCAGGCCAACGTCTGGCTCTTTCTCAAGCTCTCC
133      G  A  S  S  G  L  V  H  F  Q  I  S  K  E  G  S  D  L  S  L  I  E  Q  A  N  V  W  L  F  L  K  L  S
702     AAGACCAACAGAAGCCGCGCCAAAGTGACCATACAGCTGTACCAGAAAAAGCAAGGCACGGCAAAAGGGGAAGAAGAGCTGGTCGTCTCAGAGAAAGCT
166      K  T  N  R  S  R  A  K  V  T  I  Q  L  Y  Q  K  K  Q  G  T  A  K  G  E  E  E  L  V  V  S  E  K  A
801     GTGGACACGCGGAGGAGTGGCTGGCACACCCTGCCAGTCTCCAGCAGTGTCCAGGCACTGCTGGAAAAGGGACACAACTCCCTGGATCTGCGGATAGCC
199      V  D  T  R  R  S  G  W  H  T  L  P  V  S  S  S  V  Q  A  L  L  E  K  G  H  N  S  L  D  L  R  I  A
900     TGTGAGCAGTGTCAGGAGGCCGGCGCCACCCCTATCCTCGTAGAAAAGGATGAGCGGGAGCAGTCTCACCGGCCCTTCCTCATGCTGGCGATACGACAG
232      C  E  Q  C  Q  E  A  G  A  T  P  I  L  V  E  K  D  E  R  E  Q  S  H  R  P  F  L  M  L  A  I  R  Q
999     TCTGATGAGCAGCCCCACCGGCGGAGAAAAAGGGGCCTGGAATGCGATGGAAAGATCAGCATCTGCTGCAAGAGACAGTTTCAAGTCAGCTTCAAGGAC
265      S  D  E  Q  P  H  R  R  R  K  R  G  L  E  C  D  G  K  I  S  I  C  C  K  R  Q  F  Q  V  S  F  K  D
1098    ATCGGCTGGAACGACTGGATTATAGCCCCCACGAGCTACCACGCCAACTATTGTGAAGGGGACTGCCCAAGCCACATAGCGGGGTCCTCTGGCACCTCT
298      I  G  W  N  D  W  I  I  A  P  T  S  Y  H  A  N  Y  C  E  G  D  C  P  S  H  I  A  G  S  S  G  T  S
1197    CTCTCCTTTCACTCGACTGTCATCAACCATTACAGGATGCGGGGTATCAGCCCCTTTAACAACATCAAGTCCTGCTGCGTGCCCACTAAACTACGGGCC
331      L  S  F  H  S  T  V  I  N  H  Y  R  M  R  G  I  S  P  F  N  N  I  K  S  C  C  V  P  T  K  L  R  A
1296    ATGTCCATGTTGTACTACGACGACGGGCAGAACATTATTAAAAAGGATATGCAGAACATGATAGTAGAGGAATGTGGCTGCTCTTAAAGACAAGCAGAC
364      M  S  M  L  Y  Y  D  D  G  Q  N  I  I  K  K  D  M  Q  N  M  I  V  E  E  C  G  C  S  *
1395    TGCCCTGAACATGTAAAGGACATGGTAGCTGGGGGAAAGACATTCCTCCCTTTCCTCCAACAGACCCACAAACCTTGACAGACACAGCATGTAGTTAAA
1494    TTCATAGTGTGCTGGTTACCAGTCACTTGGACCAGTCTGTGGCACTTTCAGAAAAAAAAAAAAAAAAAAAAAAAAAAAA
```
